# Two Extension Block Kirschner Wires' Technique for Bony Mallet Thumb

**DOI:** 10.1155/2016/8086594

**Published:** 2016-09-27

**Authors:** Yutaka Mifune, Atsuyuki Inui, Fumiaki Takase, Yasuhiro Ueda, Issei Shinohara, Ryosuke Kuroda, Takeshi Kokubu

**Affiliations:** Department of Orthopaedic Surgery, Kobe University Graduate School of Medicine, Kobe 650-0017, Japan

## Abstract

Mallet fingers with an avulsion fracture of the distal phalanx or rupture of the terminal tendon of the extensor mechanism is known as a common injury, while mallet thumb is very rare. In this paper, the case of a 19-year-old woman with a sprained left thumb sustained while playing basketball is presented. Plain radiographs and computed tomography revealed an avulsion fracture involving more than half of the articular surface at the base of the distal phalanx. Closed reduction and percutaneous fixation were performed using the two extension block Kirschner wires' technique under digital block anesthesia. At 4 months postoperatively, the patient had achieved excellent results according to Crawford's evaluation criteria and had no difficulties in working or playing basketball. Various conservative and operative treatment strategies have been reported for management of mallet thumb. We chose the two extension block Kirschner wires' technique to minimize invasion of the extensor mechanism and nail bed and to stabilize the large fracture fragment.

## 1. Introduction

Injury to the tip of a finger, such as an avulsion fracture of the distal phalanx or a rupture of the terminal tendon of extensor mechanism, often occurs during sports activity [1]. However, such injuries to the thumb are rare, and mallet thumb with a dorsal avulsion fracture of the distal phalanx, that is, bony mallet thumb, is extremely rare [2]. A case of bony mallet thumb is presented here. The fracture was repaired using the two extension block Kirschner wires' technique, and the patient achieved excellent functional recovery.

## 2. Case Presentation

**Figure 1:**
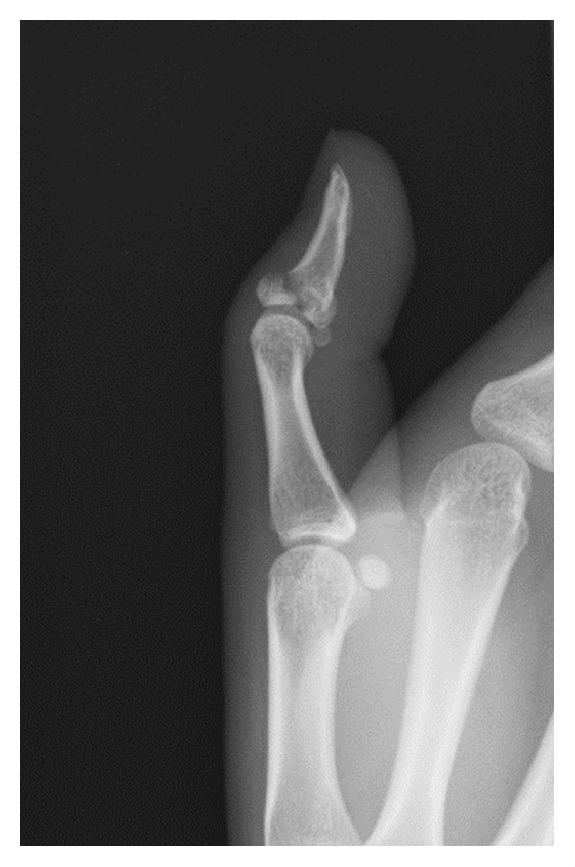
The plain radiograph shows a bony mallet thumb.

**Figure 2:**
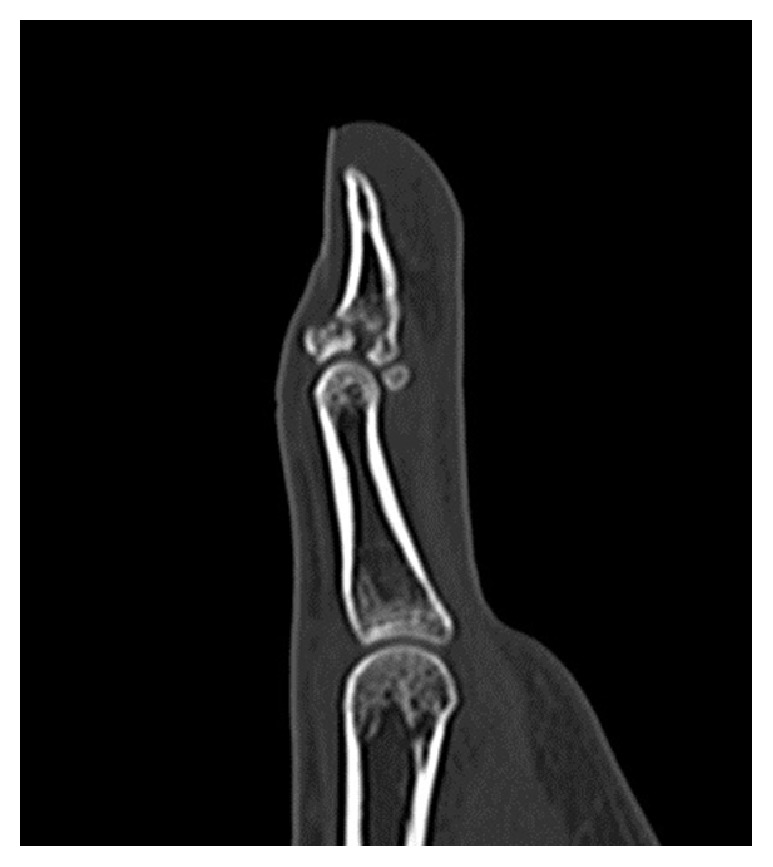
Computed tomography shows an avulsion fracture at the base of the distal phalanx involving more than half of the articular surface.

**Figure 3:**
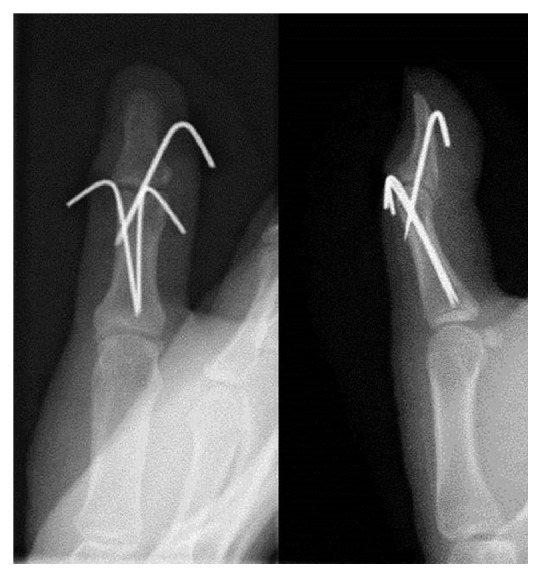
The fracture was repaired by the two extension block Kirschner wires' technique.

**Figure 4:**
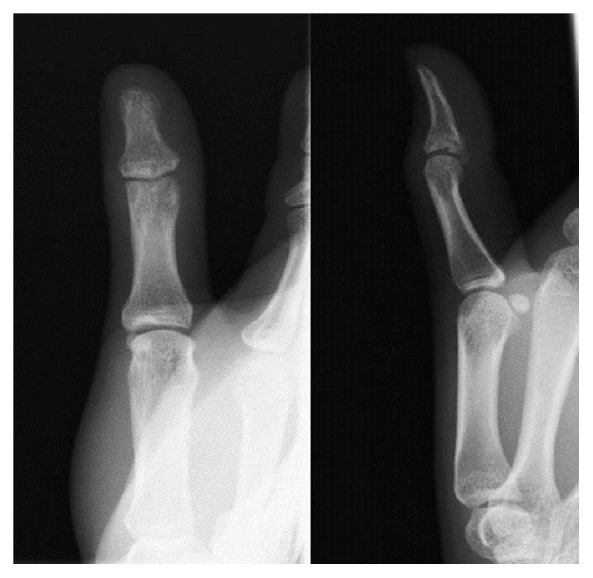
The plain radiograph at 3 months after surgery shows bony union of the avulsion fracture.

A 19-year-old woman sprained her left thumb while playing basketball and presented with tenderness and swelling around the dorsal side of the interphalangeal (IP) joint. On physical examination, she could not actively extend the joint but neither passive extension nor active flexion of the joint were disturbed. There was no instability of the IP joint against radial and ulnar stress. Radiographs and computed tomography showed an avulsion fracture at the base of the distal phalanx involving more than half of the articular surface (Figures 1 and 2). A reduction maneuver was performed but sufficient reduction could not be achieved. Therefore, closed reduction and percutaneous fixation were performed using the two extension block Kirschner wires' technique under digital block anesthesia [3]. Two 1.2 mm Kirschner wires were used for the extension block and one 1.5 mm wire was used for immobilization of the IP joint (Figure 3). All wires were removed 4 weeks after the operation, after which active flexion of the IP joint was allowed. Four months after surgery, radiographs showed bony union of the avulsion fracture (Figure 4), and the active range of motion of the IP joint was 80 degrees on flexion and −5 degrees on extension (the range of motion on the contralateral side was 90 degrees on flexion and 0 degrees on extension). According to Crawford's evaluation criteria [4], these results were excellent and the patient had no difficulties working or playing basketball.

## 3. Discussion

Mallet finger due to avulsion fracture of the distal phalanx or rupture of the terminal tendon of the extensor mechanism is known as a common injury, but mallet thumb is rare. A series by Robb included mallet deformity of 149 digits, only one of which was a mallet thumb [5], and a series by Wehbe and Schneider included only one mallet thumb among mallet fractures of 160 digits [2]. The thumb is shorter than the fingers and the extensor pollicis longus (EPL) is thicker than the extensors of the fingers, which could be one of the reasons for the rarity of EPL avulsion or laceration [6].

Because of the rarity of mallet thumb, there is no consensus regarding the recommended treatment. Din and Meggit published a report that included 4 cases of mallet thumb without avulsion fracture and showed excellent results with operative treatment [7], while McCarten et al. reported a series of 4 cases of mallet thumb in which all were conservatively treated only by 8-week immobilization of the IP joint, resulting in satisfactory outcome [8]. Crawford reported 3 cases of closed mallet thumb without fracture, 2 of which were treated with reattachment using a suture anchor and postoperative thumb spica cast immobilization, resulting in rapid recovery of complete function [4].

Various surgical treatments have been advocated for bony mallet fingers including tension band fixation, pull-in suturing, extension block with Kirschner wires, hook plate, external fixation, compression pins, and biodegradable device, with excellent results [3, 9–14]. However, unlike open injuries or injuries with a large bony fragment and subluxation of the IP joint, the indication for surgery in closed acute mallet finger remains controversial [15]. Fractures involving 30 to 50% of the joint surface are regarded as unstable and require surgical reduction to prevent subluxation of the distal phalanx fracture fragment [2, 16]. Furthermore, it has been reported that separation of bone fragments indicates periosteal laceration and that inadequate reduction could cause degenerative arthritis [17]. In the present case, radiographs and computed tomography showed an avulsion fracture involving more than half of the articular surface with separation of the bone fragment. Therefore, we performed surgical reduction and fixation. Ishiguro's method [1] is reported to be easier than open surgery. However, if the dorsal fragment is large, it is not easy to control the fragment indirectly with a single Kirschner wire. Recently, a modified extension block Kirschner wire technique that uses two extension block wires has been reported [18]. Anatomical reduction is obtained more easily with two extension block wires, and the parallel wires make contact with a wide area of the fragment, which is strongly compressed upon extension of the distal phalanx, thereby achieving and maintaining a better reduction. Because the dorsal fragment was large in our case, we selected the two-wire technique to maintain the reduction of the large bone fragment. In this case, we performed a computed tomography to make an accurate measurement of the fracture fragment width, which could help us to decide the position of two extension block Kirschner wires.

As a limitation, the follow-up period of this case was short; therefore, further follow-up will be needed for the long-term result.

In conclusion, we encountered a rare case of bony mallet thumb that we repaired using the two extension block Kirschner wires' technique and the outcome was excellent.
